# Pseudo-Malignancy: A Defensible Neologism?

**Published:** 1983-10

**Authors:** J. D. Davies

**Affiliations:** Dept. of Pathology, The Medical School, Bristol


### Correspondence

### Pseudo-malignancy: A Defensible Neologism?

Sir

Certain words ought to exist but officially do not. I remember vividly the unsuccessful attempts of a 15-year old classmate trying to convince a sceptical schoolmaster that the English language was deficient in not possessing the contraction 'Anyet' to stand in place of 'And yet'. There must have been anarchy brewing in that class, for I should now like to suggest that the Oxford English Dictionary and its subsequent supplements could be thought deficient in not allowing a line or two for 'pseudo-malignancy'.

Clinicians are evidently allowed 'pseudo-tumours',[1] and even pathologists may write of 'pseudo-epitheliomatous hyperplasias',[2] or 'pseudo-lymphomas'.[3] Alas, there currently does not seem to be a more general term to describe infiltrative or cytologically atypical lesions which, although microscopically worrisome, are not clinically progressive or even histological neoplasms. Such lesions occur in many organs,[4] may produce clinically and radiologically obvious manifestations, and yet, are neither neoplasms nor malignant. Their diagnosis is microscopic and an explanation of their nature would be facilitated by the use of the term 'pseudo-malignancy'.

If, Sir, the readers of your columns were to be offended, or potentially confused, by the use of this neologism perhaps they could be given the opportunity to chide the author of this letter. Maybe he has no better case than his classmate in attempting to promote the cause of yet another candidate for orthodox English usage.

J. D. Davies
Dept. of Pathology,
The Medical School,
Bristol

### REFERENCES

1. JEFFERSON, A. A. (1983) Benign intracranial hypertension *in* Oxford Textbook of Medicine. Editors D. J. Wetherall, J. G. G. Ledingham, D. A. Warrell. Oxford University Press, Oxford, p. 21, 81.
2. EVANS, R. W. (1966) Histological appearances of tumours. E. & S. Livingstone, Edinburgh, 2nd Edition, p. 888.
3. A supplement to the Oxford English Dictionary (1982) Editor R. W. Burchfield. Volume III: O-Scz, Clarendon Press, Oxford, pp. 872 and 875.
4. HAMPERL, H. (1975) Strahlige Narben und obliterierende Mastopathie. *Virchows Archiv. A. (Path. Anat. and Histol.)* **369**, 55–68.

